# Notes and Notices

**Published:** 1893-11

**Authors:** 


### NOTES AND NOTICES.

Dr. A. Quackenbush has succeeded to the practice of Dr. Cook, in Belleville, Ontario, Can.

Dr. F. H. Krebs has removed his office to 42 Union Park, Boston. Consultation hours, till 11 A. M., 3 to 5 P. M.; Sundays, till 11 A. M.

---

### FUN FOR DOCTORS.

Cause and Effect.—Undertaker (to Harlem physician)—"Did a stranger call on you to day for treatment?"

Physician—"No."

"That's strange. The gentleman was looking for a physician, and I recommended you very highly."

"Yes, I guess that's the reason he didn't come to see me."—*Texas Siftings.*

Servant (to his master, a very young doctor, who is at a banquet)—"Come home quickly, sir. There's a patient waiting for you. (Aside) I've locked the office door on him so that he can't escape."—*Fliegende Blaetter.*

